# Accidental intoxications in toddlers: lack of cross-reactivity of vilazodone and its urinary metabolite M17 with drug of abuse screening immunoassays

**DOI:** 10.1186/s12907-019-0084-9

**Published:** 2019-02-18

**Authors:** Christina D. Martinez-Brokaw, Joshua B. Radke, Joshua G. Pierce, Alexandra Ehlers, Sean Ekins, Kelly E. Wood, Jon Maakestad, Jacqueline A. Rymer, Kenichi Tamama, Matthew D. Krasowski

**Affiliations:** 1 Department of Chemistry, College of Sciences, NC State University, Raleigh, NC 27695 USA; 2 Comparative Medicine Institute, NC State University, Raleigh, NC 27695 USA; 3 Department of Emergency Medicine, University of Iowa Hospitals and Clinics, Iowa City, Iowa 52242 USA; 4 Department of Pathology, University of Iowa Hospitals and Clinics, 200 Hawkins Drive, Iowa City, IA 52242 USA; 5 Collaborations Pharmaceuticals, Inc., 840 Main Campus Drive, Lab 3510, Raleigh, NC 27606 USA; 6 Stead Family Department of Pediatrics, University of Iowa Stead Family Children’s Hospital, Iowa City, Iowa 52242 USA; 7 Clinical Laboratories, University of Pittsburgh Medical Center Presbyterian Hospital, Pittsburgh, PA USA; 8 Department of Pathology, University of Pittsburgh School of Medicine, Pittsburgh, PA USA; 9 McGowan Institute for Regenerative Medicine, University of Pittsburgh, Pittsburgh, PA USA; 10 Clinical Laboratory, Children’s Hospital of Pittsburgh of UPMC, Pittsburgh, PA USA

**Keywords:** Amphetamines, False positive reactions, Immunoassay, Similarity, Toxicology

## Abstract

**Background:**

Vilazodone is an FDA approved medication used to treat major depressive disorder. The authors describe two cases of accidental vilazodone exposure in toddlers who presented with symptoms similar to amphetamine exposure and also with unexplained positive amphetamine urine immunoassay drug screens. Given a lack of published data on cross-reactivity of vilazodone and its metabolites with drug of abuse screening tests, the authors investigated drug of abuse immunoassay cross-reactivity of vilazodone and metabolites using computational and empirical approaches.

**Methods:**

To ascertain the likelihood that vilazodone would cross-react with drug of abuse screening immunoassays, the authors assessed the two-dimensional (2D) similarity of the vilazodone parent molecule and known metabolites to an array of antigenic targets for urine immunoassay drug screens. To facilitate studies of the commercially unavailable M17 metabolite, it was prepared synthetically through a novel scheme. Urine and serum were spiked with vilazodone and M17 into urine (200–100,000 ng/mL) and serum (20–2000 ng/mL) samples and tested for cross-reactivity.

**Results:**

Computational analysis using 2D similarity showed that vilazodone and metabolites have generally low similarity to antigenic targets of common drug of abuse screening immunoassays, predicting weak or no cross-reactivity. The M17 metabolite had 2D similarity to amphetamines and tricyclic antidepressants in a range similar to some other compounds exhibiting weak cross-reactivity on these immunoassays. Cross-reactivity testing was therefore performed on two different urine amphetamines immunoassays and a serum tricyclic antidepressant immunoassay. However, actual testing of cross reactivity for vilazodone and the M17 metabolite did not detect cross-reactivity for any urine amphetamines screen at concentrations up to 100,000 ng/mL and for a serum tricyclic antidepressants assays at concentrations up to 2000 ng/mL.

**Conclusion:**

While the vilazodone metabolite M17 has weak 2D structural similarity to amphetamines and tricyclic antidepressants, the current study did not demonstrate any experimental cross-reactivity with two different urine amphetamines immunoassays and a serum tricyclic antidepressant immunoassay. Vilazodone ingestions in young children present a diagnostic challenge in their similarity to amphetamine ingestions and the lack of routine laboratory tests for vilazodone. Further work is needed to understand the metabolic profile for vilazodone in children versus adults.

**Electronic supplementary material:**

The online version of this article (10.1186/s12907-019-0084-9) contains supplementary material, which is available to authorized users.

## Background

Vilazodone is a medication that is used to treat major depressive disorder [1, 2]. Vilazodone was approved by the United States Food and Drug Administration in 2011 and is a selective serotonin reuptake inhibitor (SSRI) that also has partial serotonin (5-hydroxytryptamine; 5-HT) agonist activity at the 5-HT_1A_ receptor [3]. Efficacy and tolerability in adult patients appear to be similar to other SSRIs [2]. Vilazodone is marketed for adult patients, and there are no published studies of metabolism, pharmacokinetics or clinical efficacy of vilazodone in children.

While overdose data for vilazodone is limited, toxic effects appear to be similar to effects seen with other SSRIs [2]. The medication reaches peak serum concentration 4 to 5 h after ingestion [4]. The most commonly reported effects in overdose are drowsiness, vomiting, tachycardia, and agitation [5–12]. Seizures and serotonin syndrome have been reported in accidental pediatric ingestions [5, 6, 9, 10]. The United States National Poison Data System contained 753 reports of vilazodone ingestions in children younger than 6 years of age from 2011 through 2016 [8]. Overall, tachycardia, agitation, tremor, and seizures (or seizure-like activity) appear to be more common with accidental vilazodone poisonings in young children as compared with similar ingestions of other SSRIs [8, 12].

**Fig. 1:**
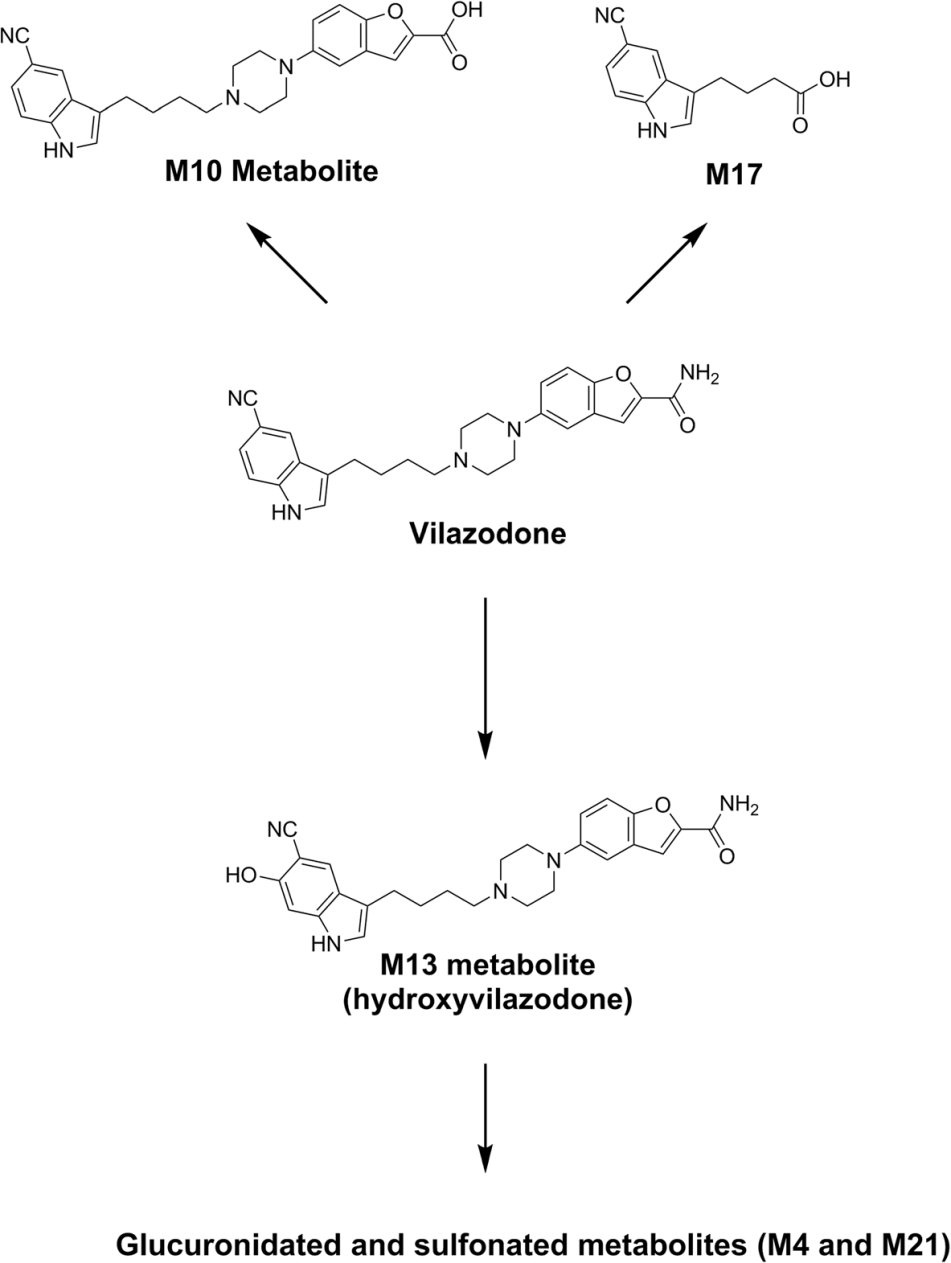
Metabolic Pathways of Vilazodone. Information compiled from multiple sources [4, 13–15]

Vilazodone has a complicated metabolic pathway in humans and other mammals [4, 13–15]. To date, vilazodone pharmacokinetic studies have only been done in adults. Two of the main metabolites in human urine have been designated M10 and M17 [14]. M10 is the carboxylic acid derivative of vilazodone, while M17 is the butyric acid of the indole fragment of the *N*-dealkylation product of vilazodone (Fig. 1). Additional metabolites include M13 (6-hydroxyvilazodone), the 5-cyano-6-hydroxy indole metabolite of vilazodone [14]. M13 is further modified by glucuronidation or sulfation of the 6-hydroxyurea moiety. While vilazodone and the M10 and M13 metabolites are identical in chemical structure except for one functional group, M17 is much more distinct, being a smaller fragment and modification of the vilazodone structure.

In previous publications, we reported case series of accidental ingestions of amphetamine and other drugs associated with similar clinical signs and symptoms on overdose. This also included retrospective analysis of potential causes of amphetamine positive immunoassay screens [16, 17]. Vilazodone was identified as a drug associated with unexplained positive amphetamine urine immunoassay drug screens in 2 toddlers. We found no published data, either in journal articles or assay package inserts, on vilazodone or vilazodone metabolite cross-reactivity with drug of abuse immunoassay screening tests. In addition, we found no commercial sources for any of the recognized vilazodone metabolites.

We thus investigated whether vilazodone and metabolites were likely to produce cross-reactivity on drug of abuse immunoassay screens using two main approaches. First, we utilized computational two-dimensional (2D)-similarity methods to compare the structural similarity of vilazodone and its recognized metabolites to the antigenic targets of urine immunoassay screens including amphetamines. We have previously used this methodology for the prediction of cross-reactivity of compounds to drug of abuse screening and therapeutic drug monitoring assays [18–22]. In the present study, these methods identified the M17 metabolite as potentially weakly cross-reactive with amphetamines and tricyclic antidepressant (TCA) immunoassays, although with relatively low 2D similarity compared to known cross-reactive compounds for these assays. Because M17 was not commercially available, we developed a novel synthetic scheme for this compound and report the synthetic details in this report along with cross-reactivity testing. Second, we tested vilazodone and the M17 metabolite for cross-reactivity on two different urine amphetamines screens and also a serum TCA immunoassay.

## Methods

### Institutional setting and electronic medical record review

University of Iowa Hospitals and Clinics (UIHC) is a 761 bed tertiary/quaternary care academic medical center located in Iowa City, Iowa. As described in our previous studies, Epic Reporting Workbench (RWB) search functions were used to identify patients from data in the electronic medical record (EMR) based on specific parameters [23]. RWB search queries identified patients who had urine amphetamine screening performed and who were known to be prescribed vilazodone. RWB queries also interrogated problem lists and diagnosis codes for drug overdoses to identify any additional vilazodone ingestions treated at UIHC.

### 2D molecular similarity analysis

Comparison of similarity of test molecules to the target compounds of drug of abuse screening immunoassays used 2D similarity analysis, which determines the similarity between molecules independent of any in vitro data [24–26]. We have applied these methods in previous publications on cross-reactivity of drug of abuse screening and other immunoassays [18, 19, 21, 22, 27]. 2D similarity searching used the “find similar molecules by fingerprints” protocol in Discovery Studio version 3.5 (Biovia, San Diego, California, USA). MDL public keys (a specific 2D similarity algorithm) were used with an input query and with the Tanimoto similarity coefficient as the output (the coefficient ranges from 0 to 1, with 1 being maximally similar and 0 being maximally dissimilar; a comparison of a compound with itself or to a very closely related molecule can produce an output of 1). 2D similarity for each test compound was compared to the target molecule of the immunoassay. We compared the 2D similarities to our previous studies modelling immunoassay cross-reactivity [18, 19, 21, 22].

### Chemical synthesis of Vilazodone metabolite M17

**Fig. 2:**
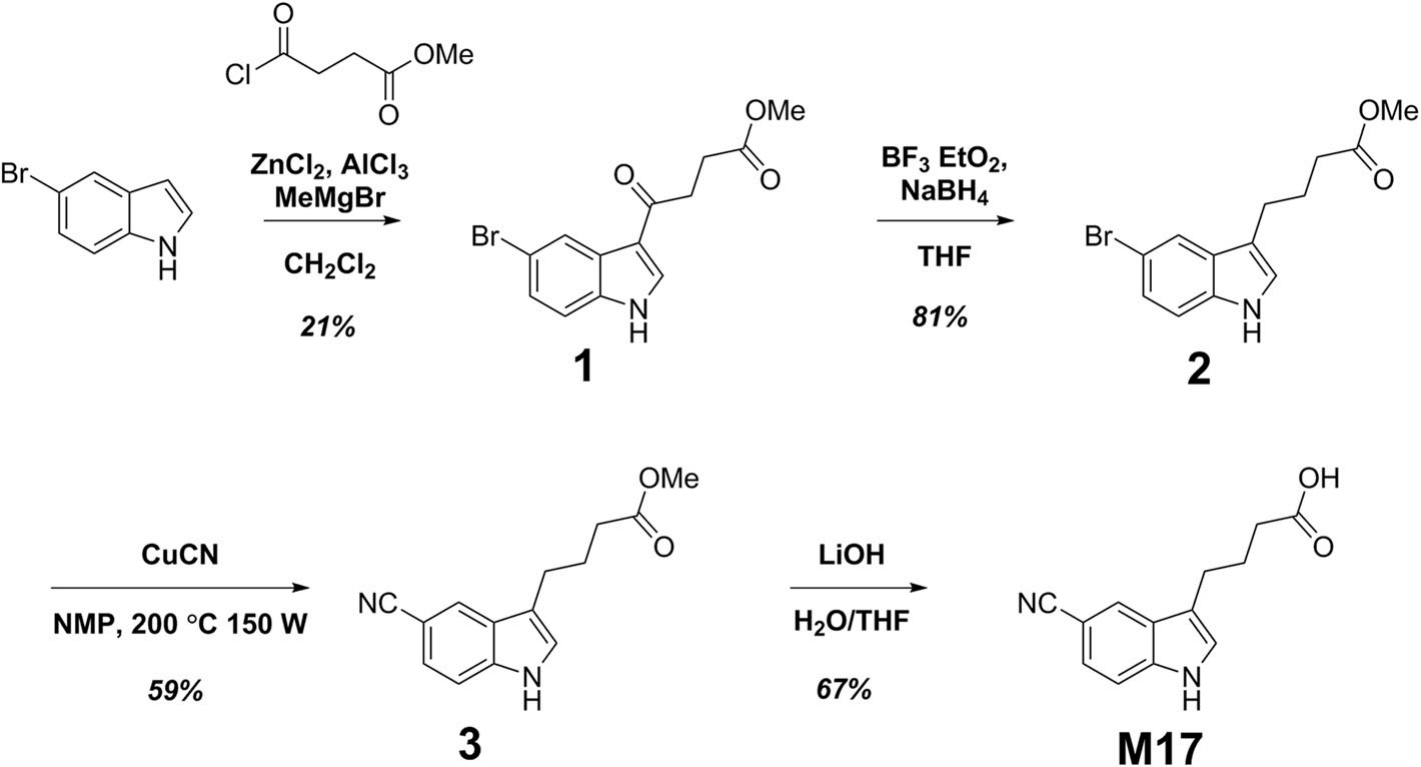
Chemical Synthetic Scheme for the M17 Metabolite of Vilazodone

The synthesis scheme for the vilazodone metabolite M17 is summarized in Fig. 2, and the detailed procedures and characterization data can be found in Additional File 1.

### Cross-reactivity studies

As detailed in the Results section, 2D molecular similarity analysis revealed potential weak cross-reactivity of the vilazodone M17 metabolite with the targets of amphetamines and TCA immunoassays. We therefore tested the vilazodone parent drug (Sigma-Aldrich, St. Louis, MO) and M17 metabolite on two different urine amphetamines immunoassays – Roche Diagnostics (Indianapolis, IN) Amphetamines II Assay (version 9.0) run on cobas c502 analyzer and Siemens Syva Emit II plus Amphetamines Assay (version 2013–07) run on a Viva-E analyzer (Siemens Diagnostics, Tarrytown, NY). We also tested the compounds on the Roche Diagnostics Benzodiazepines Plus (version 10.0), Cocaine II (version 7.0), Opiates II (version 11.0), Oxycodone (version 7.0), and Cannabinoids II (version 9.0). Serum TCA screening was performed with the Syva Emit tox Serum Tricyclic Antidepressants Assay (version 2012–06) run on a Viva-E analyzer.

Cross-reactivity testing was performed as previously described [21] up to a concentration of 100,000 ng/mL spiked in drug-free urine (200 ng/mL, 2000 ng/mL, 10,000 ng/mL, and 100,000 ng/mL). We found no prior literature on urine vilazodone concentrations but, as described below, case #1 had a urine vilazodone concentration of 120 ng/mL. We tested up to 100,000 ng/mL for vilazodone and the metabolite M17 as this covers approximately three orders of magnitude beyond the 120 ng/mL and was the highest feasible concentration given limited availability of the custom-synthesized M17 compound.

Published pharmacokinetic studies of vilazodone in adults show maximum serum/plasma concentrations of approximately 150 ng/mL or below for vilazodone and M17 [4, 13]. A report of two children who experienced seizures following accidental vilazodone ingestion reported serum concentrations of vilazodone [7]. A 3 year old boy who ingested up to seven 40-mg tablets from a foil pack had serum vilazodone concentrations of 1600 ng/mL and 360 ng/mL, respectively, from samples drawn 4 and 27 h after ingestion. A 28 month old boy who ingested a single 40 mg tablet had a serum concentration of 370 ng/mL in a sample drawn 4 h after ingestion. For serum TCA testing, compounds were spiked into drug-free serum at concentrations up to 2000 ng/mL (20 ng/mL, 200 ng/mL, and 2000 ng/mL) to cover the range of serum concentrations observed in these studies.

## Results

### Case histories of toddlers with accidental vilazodone ingestions

#### Case #1

A previously healthy 2 year old boy was noted by his father at 2200 to have a “blank stare”, clenched teeth, and unresponsiveness to verbal, visual, or tactile stimuli. The patient had been normal at 1930 prior to bedtime. The patient was unable to walk and had “shaking” and restless movements of his extremities. He was taken to outside hospital (OSH) where examination showed heart rates in the 130–170 beats per minute (bpm) range, blood pressure (BP) of 110/72 mmHg, respiratory rate (RR) 30/min, body temperature 38.2 °C, and oxygen saturation of 95% on room air. On neurologic exam, he was moving all extremities but was not speaking or making eye contact.

Diagnostic testing included a complete metabolic panel, a complete blood count, urinalysis, UDS, and non-contrast computed tomography (CT) of the head. All laboratory and radiology studies were within normal limits except a result of “presumptive positive” for amphetamines on urine drug screening (UDS), with the remainder of the UDS panel (barbiturates, benzodiazepines, cocaine metabolite, opiates, phencyclidine, and tetrahydrocannabinol) being negative (Roche Diagnostics cobas 6000 system). Confirmatory amphetamine testing was not pursued. The patient was given 0.25 mg (0.02 mg/kg based on patient weight of 12.4 kg) of lorazepam for continued seizure-like activity. The presumptive diagnosis upon transfer was amphetamines intoxication.

The patient was transferred to UIHC on day #2 at 0430. On neurologic examination, his mental status was appropriate, with markedly dilated pupils responsive to light. Similar to the OSH, UDS performed at UIHC revealed a presumptive positive amphetamines screen with remainder of UDS panel (benzodiazepines, cocaine metabolite, opiates, and oxycodone/oxymorphone) negative (Roche Diagnostics cobas 8000 system). He continued to have unsteady gait with tremoring of his legs when bearing weight which steadily improved. The patient was discharged home on day #3 at 1130 with normal vital signs and physical examination.

Medications in the home included vilazodone (mother’s medication), doxylamine/vitamin B_6_, and multivitamins. Parents denied the presence of any amphetamine-containing medications in the house or other possible exposure to amphetamine or methamphetamine. Confirmatory urine amphetamines testing by liquid chromatography/tandem mass spectrometry (LC/MS/MS) was performed by reference laboratory (ARUP Laboratories, Salt Lake City, UT) and was negative for amphetamine, methamphetamine, methylenedioxyamphetamine (MDA), methylenedioxymethamphetamine (MDMA), and methylenedioxyethylamphetamine (MDEA) (lower limit of quantitation 200 ng/mL for all 5 analytes). Analysis of the urine specimen for vilazodone by LC/MS/MS (NMS Labs, Willow Grove, PA) was performed and returned a quantitative level of 130 ng/mL.

#### C*ase #2*

A 2 year old girl was noted by family members to exhibit odd behavior starting at 1130 with restlessness, flailing limbs, and rolling around on the ground. This progressed to periods of unresponsiveness where she would stare blankly. The patient had been playing while the mother was in another room around 1000. The mother noticed that the patient had taken out a pen from her purse but did not immediately suspect that she may have gotten into medications in the purse.

The patient was taken to a local emergency department at an OSH. Physical examination showed heart rates in the 150–170 bpm range, blood pressure of 106/75 mmHg, respiratory rate 30/min, body temperature 38.5 °C, and oxygen saturation of 97–100% on room air. The patient was unresponsive to verbal commands and showed involuntary movements of all extremities. Pupils were bilaterally dilated and equally reactive to light. She was given 1 mg of lorazepam (0.07 mg/kg based on weight of 14.1 kg) for the presumed seizure-like movements and transferred to UIHC for further management.

On arrival at UIHC emergency department at 1510, she was more talkative but still not at baseline mental status per family. Physical examination showed heart rates in the 130–160 bpm range, BP of 110/68 mmHg, RR 26–30/min, body temperature 37.1 °C, and oxygen saturation of 98–100% on room air. UDS revealed a presumptive positive amphetamines screen with remainder of UDS panel (benzodiazepines, cocaine metabolite, opiates, and oxycodone/oxymorphone) negative. Confirmatory urine drug testing and urine vilazodone levels were not ordered by the clinical team. On neurologic examination, the patient was more talkative and showed disorganized persistent extremity movements. These were not thought to be seizures. After examination, the patient became tired and fell asleep shortly after arrival and was back to her baseline on waking. She was discharged home on morning of day #2 with normal vital signs and physical examination.

Questioning of the family for medications that might be accessible to the child revealed that the mother’s purse contained both a bottle and foil pack of vilazodone tablets. The foil packet was missing tablets, and the mother could not recall how many tablets were there before the patient played with the purse. Further investigation revealed no likely source of amphetamines.

### Search for additional cases

Interrogation of the EMR database at UIHC did not reveal any other vilazodone ingestions in children 8 years of age or younger during the retrospective period, including in 4407 negative amphetamine screens in patients in this age range. We also searched for overlap between patients known to be actively prescribed vilazodone and positive amphetamine screens. Of 1430 adult patients (18 year or older) and 20 pediatric patients (younger than 18 years old) prescribed vilazodone and who presented at our hospital for care, none had a positive amphetamines screen during the retrospective analysis period.

### 2D similarity of vilazodone and metabolites to amphetamines

Given that only vilazodone reference standards are available commercially, studies of vilazodone metabolites for cross-reactivity require novel synthesis. To help prioritize which metabolites to pursue for novel synthesis and subsequent cross-reactivity testing, we performed computational 2D similarity analysis of vilazodone and its metabolites to the target compounds of amphetamines (Table 1). Vilazodone and the M10 and M13 metabolites each had 2D similarity to amphetamine and methamphetamine below any previously characterized compound that showed detectable cross-reactivity to amphetamines immunoassay screen (Table 1) [19, 21, 22], thus predicting low likelihood of cross-reactivity.

**Table 1:** 2D Similarity of Vilazodone and its Metabolites to Target Molecules of Drug of Abuse Immunoassays^1^

	Vilazodone	M10 metabolite	M17 metabolite	M13 (6-Hydroxyvilazodone)	Lowest 2D similarity of cross-reactive compound (previous studies)
*Amphetamines*					
Amphetamine	0.18	0.17	**0.24**	0.16	mCPP, TFMPP (0.23)^2^
Methamphetamine	0.16	0.16	**0.23**	0.14	mCPP, TFMPP (0.22) ^2^
MDMA/ecstasy	**0.33**	**0.38**	**0.37**	**0.35**	mCPP, TFMPP (0.25) ^2^
*Barbiturates*					
Secobarbital	0.39	0.36	0.28	0.42	*p*-Hydroxyphenobarbital (0.72)
*Benzodiazepines*					
Nordiazepam	0.44	0.37	0.33	0.43	α-Hydroxytriazolam (0.52)
Oxazepam	0.44	0.41	0.36	0.46	Alprazolam (0.47)
*Cannabinoids*					
Δ^9^-THC-COOH	0.25	0.29	0.27	0.30	Cannabinol (0.80)
*Cocaine metabolites* Benzoylecgonine	0.41	0.50	0.34	0.46	Cocaethylene (0.85)
*Opiates*					
Morphine	0.50	0.57	0.30	0.57	Rifampin (0.59)
Oxycodone	0.51	0.55	0.34	0.57	Noroxycodone (0.79)
Phencyclidine	0.46	0.46	0.22	0.41	Dextromethorphan (0.57)
*Synthetic opioids*					
Buprenorphine	0.50	0.57	0.33	0.58	Buprenorphine glucuronide (0.78)
Methadone	0.31	0.31	0.31	0.28	Methadol (0.86)
*Tricyclic compounds*					
Desipramine	**0.45**	**0.45**	0.33	**0.42**	Carbamazepine epoxide (0.40)
Imipramine	**0.43**	**0.44**	0.27	**0.41**	Carbamazepine epoxide (0.38)

^1^ Values in bold are cases where the 2D similarity is higher than a known cross-reactive compound reported in an assay package insert or published literature

^2^ Abbreviations: mCPP, Meta-chlorophenylpiperazine; TFMPP, Trifluoromethylphenylpiperazine

The M17 metabolite had 2D similarity to amphetamine and methamphetamine of Tanimoto coefficients of 0.24 and 0.23, respectively (scale of 0–1, with 0 being maximally dissimilar and 1 being very similar). This is only slightly higher than the lowest 2D similarity of compounds observed to be cross-reactive with amphetamines immunoassays (meta-chlorophenylpiperazine, mCPP; trifluoromethylphenylpiperazine, TFMPP); note that many compounds with this low of a 2D similarity to amphetamine or methamphetamine show no cross-reactivity and only a small fraction demonstrate weak cross-reactivity in experimental testing [19, 21, 22]. Vilazodone, M10, M13, and M17 all had low 2D similarities to MDMA/ecstasy, predicting no cross-reactivity or only by challenge with very high concentrations of the compound [21, 22].

We also compared 2D similarity of vilazodone and its metabolites to common target compounds of other drug of abuse immunoassays (barbiturates, benzodiazepines, buprenorphine, cannabinoids, cocaine metabolite, methadone, opiates, phencyclidine, and TCAs). In general, this revealed that vilazodone and its metabolites had lower 2D similarity to the target compounds of these immunoassays than compounds recognized to be cross-reactive to these assays (Table 1) [19, 21, 22]. The only exception was for the TCA immunoassay targets (desipramine and imipramine), for which vilazodone, M10, and M13 had 2D similarity slightly above that of carbamazepine epoxide, a drug metabolite with weak cross-reactivity to some marketed TCA immunoassays [21].

### Cross-reactivity testing

We therefore pursued novel chemical synthesis of M17, reasoning that it would be more likely distinct from vilazodone in immunoassay cross-reactivity testing based on the 2D similarity predictions. An overview of the chemical synthesis of M17 is provided in Fig. 2, with the detailed procedures and characterization data in Additional File 1. We tested vilazodone and its metabolite M17 for cross-reactivity to the Roche cobas 8000 urine drug immunoassays for amphetamines, benzodiazepines, cocaine metabolite, opiates, oxycodone/oxymorphone, and THC. However, we detected no cross-reactivity at concentrations up to 100,000 ng/mL for vilazodone and M17 (spiked into drug-free urine) for any of the immunoassays including amphetamines. We also did not detect any cross-reactivity for a serum TCA immunoassay at concentrations up to 2000 ng/mL.

## Discussion

There have been case reports/series and analyses of poison center reports on the signs and symptoms of vilazodone overdose in young children [5–12]. Tachycardia, agitation, tremor, and seizures were more common in poison center data with pediatric vilazodone ingestions as compared to other SSRIs [8, 12]. Ingestions in young children generally show complete recovery of function following resolution of symptoms of intoxication.

The two cases of vilazodone intoxication described in the present study caused some diagnostic confusion due to presumptive positive drug screens for amphetamines at two different hospitals (albeit by same methodology) along with clinical signs and symptoms resembling amphetamines overdose [16, 17]. Case #1 had confirmatory testing negative for amphetamines by LC/MS/MS and specialty laboratory analysis (also by LC/MS/MS) that detected vilazodone in urine, although these results came back after the child was discharged from the hospital. Case #2 occurred prior to the availability of vilazodone testing at reference laboratories. The similarity of vilazodone overdoses to amphetamines toxicity demonstrate the importance of confirmatory analysis to rule out amphetamine and methamphetamine exposure. Consideration of vilazodone ingestion in both cases only came after more detailed household history could be elicited. Analysis of vilazodone in serum or urine is not routinely available at most clinical laboratories but is performed by some specialty reference laboratories.

Accidental ingestion of amphetamines, including methamphetamine or prescription amphetamines, is common in young children, and the presenting signs and symptoms overlap to some degree with those potentially caused by vilazodone [16, 17, 28–30]. Symptoms of tremor/seizure-like movements, tachycardia, mydriasis, and agitation may also be seen with amphetamine toxicity. One clinical benefit of confirmatory testing is to help rule out child exposure to methamphetamine, which could be associated with additional risks such as living at a home illicitly manufacturing this drug.

Immunoassays for amphetamines are generally intended to optimally detect amphetamine, methamphetamine, and sometimes related drugs such as MDMA/ecstasy, MDA, and MDEA [18, 19, 21, 22, 31]. The target hapten for these assays may be amphetamine, methamphetamine, and/or MDMA, contributing to observed differences in cross-reactivity for various marketed amphetamines immunoassays [18, 21, 22]. Amphetamines assays have highly variable cross-reactivity with other structurally related compounds such as ephedrine, pseudoephedrine, phenylpropanolamine, and various ‘designer amphetamines’ such as mephedrone and methcathinone [21, 22, 32]. Amphetamines immunoassays are also subject to false positives by less obviously structurally related compounds such as 1-methyl-3-phenylpiperazine (a metabolite of the antihypertensive medication labetalol) [33–35] and mCPP (metabolite of trazodone) [36–39]. There is significant variability in testing and reporting of cross-reactive compounds, especially drug metabolites, in package inserts of commercially marketed amphetamines screening assays [40].

We investigated whether vilazodone and its major metabolites were likely to cross-react with amphetamines immunoassays using computational prediction by 2D similarity analysis. We chose 2D analysis, because we have explored 3D methods with our previous studies with amphetamines and amphetamine-like drugs but found that these do not perform well for predictions except for very closely related compounds (e.g., amphetamine, methamphetamine, MDMA) [18, 21, 22]. Among many challenges, predicting the correct molecular conformation for 3D modeling is difficult.

The 2D computational studies showed vilazodone and two metabolites (M10 and M13) were unlikely to show cross-reactivity with commonly used drug of abuse immunoassays. On the other hand, the M17 metabolite (a cleavage product of vilazodone) had some 2D similarity with amphetamines in a range in which some compounds such as mCPP and 4-chlorophenypiperazine show weak cross-reactivity with some amphetamines immunoassays. However, multiple other compounds that show no cross-reactivity to amphetamines immunoassays, including methylphenidate and labetalol (parent drug), have a similar 2D similarity profile, illustrating the challenges in predicting compounds with weak cross-reactivity [18, 22].

Our own empirical testing of the Roche Diagnostics Amphetamines II and Siemens Emit II plus Amphetamines immunoassays showed no cross-reactivity with either vilazodone or the M17 metabolite at concentrations up to 100,000 ng/mL. In addition, serum samples spiked with vilazodone or M17 up to 2000 ng/mL did not cross-react with a serum TCA immunoassay, another categories of compounds to which vilazodone has some 2D similarity. This lack of experimental cross-reactivity with urine amphetamines immunoassays suggest that the positive screens in the clinical cases are either coincidental (and caused by some other factor) or result from an additional metabolite of vilazodone not tested. We think it is unlikely that metabolites M10 or M13 cross-react with amphetamines immunoassays as these are very close in structure to vilazodone. However, it should be pointed out that the reported metabolic pathway for vilazodone has only been worked out in adults, with essentially no data on the metabolic pathway for vilazodone in young children [4, 13–15]. A number of drug-metabolizing enzymes show significant age-dependent differences in expression and functional activity [41]. There is precedence for uncharacterized metabolites in young children cross-reacting with immunoassays. Such a phenomenon has been proposed to explain positive THC immunoassay screens in newborns with known exposure to cannabis in utero, where the immunoassay screen is positive but with a mass spectrometry-based THC confirmatory assay negative for THC metabolites common in adults [42].

## Conclusions

In summary, vilazodone and its M17 metabolite did not demonstrate cross-reactivity with amphetamines, tricyclic antidepressants, or other common drug of abuse screening immunoassays. Healthcare professionals and clinical laboratories should be aware of the similarities in the clinical presentations of vilazodone and amphetamine toxicity in children and utilize confirmatory testing when indicated. Future studies can also aim to elucidate the metabolism profile for vilazodone in children versus adults, including in overdose situations.

## **Additional file**

Additional file 1: Chemical Synthesis and Characterization of Vilazodone Metabolite M17. (DOCX 27 kb)

## Abbreviations

2D: Two-dimensional
5-HT: Serotonin or 5-hydroxytryptamine
LC/MS/MS: Liquid chromatography-tandem mass spectrometry
mCPP: Meta-chlorophenylpiperazine
MDMA: 3,4-methylenedioxymethamphetamine (ecstasy)
SSRI: Selective serotonin reuptake inhibitor
TFMPP: Trifluoromethylphenylpiperazine
UDS: Urine drug screening
