# Supplementary material for: Accidental intoxications in toddlers: lack of cross-reactivity of vilazodone and its urinary metabolite M17 with drug of abuse screening immunoassays
Source: BMC Clin Pathol. 2019 Feb 18;19:2. doi: 10.1186/s12907-019-0084-9 (PMC6379996; doi:10.1186/s12907-019-0084-9)
Supplement: Supplementary file 1 — Chemical Synthesis and Characterization of Vilazodone Metabolite M17. (DOCX 27 kb) [file 12907_2019_84_MOESM1_ESM.docx]

**Additional File 1**

**Chemical Synthesis and Characterization of Vilazodone Metabolite M17**

All reactions were performed under an Ar or N_2_ atmosphere and all glassware was dried in an oven at 125 °C overnight, unless otherwise noted. THF and CH_2_Cl_2_ were purified using an alumina filtration system. Methyl magnesium bromide, 5-bromoindole, ZnCl_2_, methyl 4-chloro-4-oxobutanoate, AlCl_3,_ NaBH_4,_ BF_3_·OEt_2_, CuCN, NMP and LiOH·H_2_O were purchased from Sigma-Aldrich or Fisher Scientific and were used without further purification. Reactions were monitored by TLC analysis (EM Science pre-coated silica gel 60 F254 plates, 250 μm layer thickness) and visualization was accomplished with a 254 nm UV light and by staining with a KMnO_4_ solution (1.5 g of KMnO_4_, 10 g of K_2_CO_3_, and 1.25 mL of a 10% NaOH solution in 200 mL of water). Reactions were also monitored by LC-MS (Shimadzu LC-MS 2020 with Kinetex 2.6 μm C18 50 x 2.10 mm). Flash chromatography on SiO_2_ was used to purify the crude reaction mixtures and performed on a Biotage Isolera utilizing Biotage cartridges and linear gradients. Infrared spectra were determined on a Jasco FT/IR-4100 spectrometer. ^1^H, ^13^C spectra were obtained on a Varian Mercury-VX 300, a Varian Mercury-VX 400, or a Varian Mercury-Plus 300 instrument in CDCl_3_ unless otherwise noted. Chemical shifts were reported in parts per million with the residual solvent peak used as an internal standard (CDCl_3_ = 7.26 ppm for ^1^H and 77.23 ppm for ^13^C). ^1^H NMR spectra were run at 300 or 400 MHz and are tabulated as follows: chemical shift, multiplicity (s = singlet, d = doublet, t = triplet, q = quartet, m = multiplet, bs = broad singlet, dt = doublet of triplet, ddd = doublet of doublet of doublet, qd = quartet of doublets), number of protons, and coupling constant(s). ^13^C NMR spectra were run at 100 MHz using a proton-decoupled pulse sequence with a d_1_ of 1 second unless otherwise noted and are tabulated by observed peak. High-resolution mass spectra were obtained on a Thermo Fisher Scientific, Exactive Plus mass spectrometer using Heated Electrospray Ionization.

**Methyl 4-(5-bromo-1*H*-indol-3-yl)-4-oxobutanoate (1):** To a solution of 5-bromoindole (3.47 g, 17.7 mmol) and ZnCl_2_ (2.80 g, 18.6 mmol) in dry CH_2_Cl_2_, MeMgBr (3 M in diethyl ether, 6.20 mL, 18.6 mmol) was added dropwise under an inert atmosphere over 20 minutes at 0 °C. The reaction mixture was then warmed to room temperature and allowed to stir for 30 minutes before the addition of methyl 4-chloro-4-oxobutanoate (2.80 g, 2.29 mL, 18.6 mmol). AlCl_3_ (1.18 g, 8.85 mmol) was slowly added and the reaction was monitored by TLC. Upon full consumption of 5-bromoindole, the reaction was quenched with saturated NH_4_Cl (aq) (50 mL) and transferred to a separatory funnel containing H_2_O (20 mL) and CH_2_Cl_2_ (50 mL). The aqueous layer was further extracted with CH_2_Cl_2_ (4 x 100 mL), the organic layers combined, washed (sat. aq. NaHCO_3_, 50 mL), brine (50 mL) and dried (Na_2_SO_4_). The crude solution was then concentrated *in vacuo* and purified by recrystallization (CH_2_Cl_2_) to yield **1** as a colorless solid (1.23 g, 21%): ^1^H NMR (300 MHz, DMSO) δ 12.20 (brs, 1 H), 8.44 (s, 1 H), 8.27 (s, 1 H), 7.45 (d, 1 H, *J* = 8.6 Hz), 7.34 (d, 1 H, *J* = 8.57 Hz), 3.59 (s, 3 H), 3.18 (t, 2 H, *J* = 6.5 Hz ), 2.64 (t, 2 H, *J* = 6.5 Hz); ^13^C NMR (100 MHz, DMSO) δ 193.7, 173.5, 135.7, 135.4, 127.5, 125.8, 123.8, 114.9, 114.7, 51.8, 33.9, 28.2; ESIMS *m/z* 310 [M+H]^+^.

**Methyl 4-(5-bromo-1*H*-indol-3-yl)butanoate (2):** To a stirred solution of (**1)** (937 mg, 3.02 mmol) in dry THF (10 mL), NaBH_4_ (229 mg, 6.04 mmol) was added and the reaction was allowed to stir for 20 minutes under an inert atmosphere. BF_3_·OEt_2_ (1.29 g, 1.12 mL, 9.06 mmol) was slowly added and the reaction was monitored by HPLC-MS. Upon completion, the reaction was carefully quenched with H_2_O (10 mL) and extracted with EtOAc (3 x 25 mL). The combined organic layers were dried (Na_2_SO_4_) and concentrated *in vacuo*. The crude residue was purified by column chromatography (hexanes:EtOAc, 10 to 100%) to yield **2** as a colorless oil (725 mg, 81%): ^1^H NMR (300 MHz, CDCl_3_) δ 8.02 (brs, 1 H), 7.71 (s, 1 H), 7.20-7.27 (m, 2 H), 6.99 (s, 1 H), 3.67 (s, 3 H), 2.75 (t, 2 H, *J* = 7.4 Hz), 2.38 (t, 2 H, *J* = 7.4 Hz), 2.02 (p, 2 H, *J* = 7.9 Hz); ^13^C NMR (100 MHz, CDCl_3_) δ 174.2, 135.0, 129.2, 124.7, 122.7, 121.5, 115.3, 112.5, 112.4, 51.5, 33.6, 25.2, 24.3; ESIMS *m/z* 310 [M+H]^+^.

**Methyl 4-(5-cyano-1H-indol-3-yl)butanoate (3):** CuCN (39.5 mg, 0.441 mmol) and (**2**) (100 mg, 0.339 mmol) in 0.5 mL of NMP was submitted to microwave irradiation (200 ˚C, 150 W) for 15 minutes. The reaction mixture was then diluted with EtOAc (5 mL) and filtered before washing with H_2_O (3 x 5 mL). The organic layer was dried (MgSO_4_) and concentrated *in vacuo* to yield a crude residue which was purified by column chromatography (hexanes:EtOAc, 10 to 100%) to afford **3** as an light-yellow oil (47 mg, 59%): ^1^H NMR (300 MHz, CDCl_3_) δ 8.26 (brs, 1 H), 7.96 (s, 1 H), 7.41 (dd, 2 H, *J* = 8.4, 7.9 Hz), 7.12 (s, 1 H), 3.67 (s, 3 H), 2.89 (t, 2 H, *J* = 7.6 Hz), 2.39 (t, 2 H, *J* = 7.4 Hz), 2.04 (p, 2 H, *J* = 7.5 Hz); ^13^C NMR (100 MHz, CDCl_3_) δ 173.9, 137.9, 127.4, 125.0, 124.7, 123.5, 120.8, 116.8, 111.9, 102.5, 51.6, 33.5, 25.2, 24.2; HRMS m/z calculated for C_14_H_14_N_2_O_2_ [M+H]^+^ 243.11280, found 243.11217.

**4-(5-Cyano-1H-indol-3-yl)butanoic acid (4):** To a 1:1 THF/H_2_O (0.3 mL), ester **3** (13 mg, 0.054 mmol) and LiOH·H_2_O (33 mg, 0.81 mmol) was added. The mixture was stirred for 12 h before diluting with H_2_O (5 mL) and acidification with 3N HCl. Extraction with EtOAc (3 x 5 mL) and concentration *in vacuo* afforded **4** as a light-yellow oil (8 mg, 67%): ^1^H NMR (300 MHz, CD_3_OD) δ 7.98 (s, 1 H), 7.46 (d, 1 H, *J* = 8.5 Hz), 7.35 (d, 1 H, *J* = 10.0 Hz), 7.21 (s, 1 H), 2.81 (t, 2 H, *J* = 7.5 Hz), 2.35 (t, 2 H, *J* = 7.3 Hz), 1.99 (p, 2 H, *J* = 7.4 Hz); ^13^C NMR (100 MHz, CD_3_OD) δ 176.0, 138.5, 127.3, 124.3, 123.9, 123.6, 120.6, 115.7, 111.9, 100.6, 33.0, 25.4, 23.7; IR (neat) 3336, 2928, 2858, 2097, 1670, 1523 cm^-1^; HRMS m/z calculated for C_13_H_12_N_2_O_2_ [M+H]^+^ 229.09715, found 229.09707.
